# Acute effect of complexity in basketball on cognitive capacity

**DOI:** 10.3389/fpsyg.2024.1376961

**Published:** 2024-05-16

**Authors:** Alejandro Gutiérrez-Capote, Iker Madinabeitia, Francisco Alarcón, Elisa Torre, Jesús Jiménez-Martínez, David Cárdenas

**Affiliations:** ^1^Department of Physical Education and Sport, Faculty of Sports Science, University of Granada, Granada, Spain; ^2^Sport and Health University Research Institute (iMUDS), Granada, Spain; ^3^Department of General and Specific Didactics, Faculty of Education, University of Alicante, Alicante, Spain

**Keywords:** executive function, inhibitory control, restriction, cognitive load, training, basketball

## Abstract

**Background:**

Executive functions, notably inhibition, significantly influence decision-making and behavioral regulation in team sports. However, more research must be conducted on individual player characteristics such as experience and motor skills. This study assessed how accumulated practical experience moderates inhibition in response to varying task difficulty levels.

**Methods:**

Forty-four university students (age: 20.36 ± 3.13 years) participated in this study with two sessions: one followed standard 1 × 1 basketball rules (“Regular Practice”), while the other imposed motor, temporal, and spatial restrictions (“Restriction Practice”). Functional difficulty was controlled by grouping pairs with similar skill levels. Flanker and Go-Nogo tasks were used.

**Results:**

Increasing complexity worsened cognitive performance (inhibition). “Restriction Practice” showed a significantly slower and less accurate performance in both tests than “Regular Practice” (*p* < 0.001). Experience positively impacted test speed and accuracy (*p* < 0.001).

**Conclusion:**

In sports, acute cognitive impacts are intrinsically linked to the task’s complexity and the athlete’s cognitive resources. In this sense, it is essential to adjust individually the cognitive demands of the tasks, considering each athlete’s specific cognitive abilities and capacities.

## 1 Introduction

In the broad range of definitions related to higher cognition, executive functions are identified as a group of capacities crucial for conscious and directed behavior control toward achieving specific goals (Diamond, 2013). These functions are essential for overcoming cognitive challenges in sports environments by facilitating the coordination between thought and action to achieve adaptive goals (Bravi et al., 2022). The study of the interaction between sports practice and cognitive functions promises not only to identify and enhance athletic talent (Scharfen and Memmert, 2019), but also to evaluate specific sports as potential interventions to enhance cognitive abilities in individuals with cognitive impairments, benefiting both young people and older adults (Tsai, 2009).

The inhibitory component of executive functions involves the ability to selectively concentrate on achieving a goal while ignoring distracting stimuli (Miyake et al., 2000). This component is particularly relevant in the sports domain (Liao et al., 2017), especially in interaction sports characterized by high levels of uncertainty (Swann et al., 2015), where it is crucial to control emotions and impulses. Recent research suggests that inhibition processes should be considered a set of functions rather than a single construct. Despite the various classifications proposed for the different inhibitory abilities, there is a consensus on distinguishing two main types: interference control “or perceptual inhibition” and response inhibition. The main difference between the two is that they address different manifestations of inhibitory responding: executing the response in the face of distractor stimuli (Flanker task) or not initiating any response -impulse control- (Go-Nogo) (Xie et al., 2017). Although many authors also include cognitive inhibition, such as the suppression of previously active rules in working memory in order to focus on new rules and apply them effectively, several psychometric studies suggest that this ability to change can be considered a distinct executive function (Miyake et al., 2000).

Evidence of a bidirectional interaction between inhibitory control (IC) and physical exercise (PE) exists. Most recent interventional studies (the only ones allowing causality) found a positive effect of PE on IC (Browne et al., 2016; Ludyga et al., 2019; Shigeta et al., 2021). Only one study published in the last decade failed to replicate this effect (Williams et al., 2020), and a few cases reported no effects (Lind et al., 2019). When the studies compare sports intervention with no-sport conditions, a positive effect on IC could be seen in basketball, football, and badminton (Cooper et al., 2018; Lind et al., 2019; Takahashi and Grove, 2019, 2020; Wen et al., 2021). Interaction sports are characterized by abundant dynamic and constantly changing stimuli, which demand continuous regulation and adaptation. The results of a recent meta-analysis support the cognitive stimulation hypothesis, which has been proposed to explain the different temporal effects observed on cognitive performance after a single session of various activities or sports. Albaladejo-Garcia et al. (2023) found sufficient evidence to support the notion that participation in activities that demand greater cognitive effort leads to more significant adaptations in response inhibition ability. Athletes undergo physical and mental exertion during practice that requires them to monitor several cognitive processes, including focusing attention on specific cues, ignoring distractions in their environment (Laaksonen et al., 2018), and managing relevant versus irrelevant cognitive functions (Jamro et al., 2022). This effect of PE may be mediated by different factors, such as the type of activity, both quantitative (volume, intensity, density) and qualitative (continuity of exercise, conditional quality involved, and requirements) (Gutiérrez-Capote et al., 2024).

However, some issues still need to be clarified. In contrast to the cognitive benefits of a single aerobic/sports exercise session, some studies find mental fatigue-induced impairment (Dong et al., 2022). Maintaining prolonged physical exertion hurts IC (Ferreira et al., 2024), reducing activation in the dorsolateral prefrontal cortex (Ishii et al., 2014). On the other hand, exposure to the high demands of cognitive challenge experienced by the athlete during competition may decrease his or her decision-making performance, and in which IC appears to mediate (Smith et al., 2016). Cognitive-motor interference can explain this impairment (McIsaac et al., 2018). The simultaneous performance of cognitive and motor demands to which the athlete is subjected generates competition for available neural resources, leading to decreased performance in both modalities. However, studies on the chronic effect of dual-task practice have shown increased motor and cognitive performance. A recent systematic review found an acute adverse effect of dual tasks on the cognitive performance of athletes and, in turn, a benefit after training (chronic effect) with dual tasks (Moreira et al., 2021). The lack of control and monitoring of the acute effect generated by the mental load of each session of the intervention programs to investigate its relationship with the cumulative effect over time has prevented knowing what level of stimulation is required in each session to produce the desired long-term effects. From the field of sports training, it is well known that adaptations are produced through a process of supercompensation, in which it is necessary to apply a stimulus that depletes the athlete’s resources so that the adaptive mechanisms are activated, which would generate an increase in their response capacity in such environments. An acute adverse effect on cognitive performance could activate the adaptive mechanisms to increase the athlete’s resources of this type if temporary exposure to such environments is maintained.

Therefore, we hypothesize that the mental load of the task, generated by its level of complexity, determines the positive or negative effect on subsequent cognitive performance. Previous research conducted by Gutiérrez-Capote et al. (2023) found that by introducing difficulties through practice variability and using restrictions (motor, temporal, and spatial), players experienced an increased mental load and decreased motor performance. This type of practice succeeded in keeping participants at an optimal level of challenge (Guadagnoli and Lee, 2004). Furthermore, the effects found were moderated by previous basketball experience and the inhibition ability of the starting player, both their capacity for interference and response inhibition. Based on this background, we propose as the second hypothesis of our study that the initial experience level of the subjects modulates the magnitude of the effects.

## 2 Materials and methods

### 2.1 Ethical approval

Written informed consent was obtained from all participants prior to initiating the research. This study was approved by the Ethics Committee of the University of Granada (approval number: 3616/CEIH/2023) and conducted following the guidelines established in the Declaration of Helsinki.

### 2.2 Participants

The sample size was determined from an *a priori* potential analysis (G*Power version 3.1.9.7; Faul et al., 2009) for a two-way repeated ANOVA. Analysis parameters were selected from the literature review on exercise and cognition (Verburgh et al., 2014; Ludyga et al., 2016; Booth et al., 2020). An effect size of 0.25, a power (1-β) of 0.95, an expected ICC of 0.50, and an α-level of 0.05 were set for 4 group levels with a total of 9 measures, which assumed a sample size of at least 32 participants to detect similar significant effects. Before recruiting participants, the following inclusion criteria were established: (1) be free of any documented cardiovascular, neurological, psychiatric, or mental disorders; (2) be actively engaged in physical exercise or sports activities; (3) use no medication during the study period; (4) no history of concussions in the last 30 days; and (5) be free of documented muscle and musculoskeletal injuries in the previous 3 months. Forty-four students from the Faculty of Sports Sciences, who met all the previously established inclusion criteria, were selected by email after expressing their interest in participating in the study. In order to avoid the possibility that the relationship between the study variables could be influenced by chance or the results could be moderated, participants were asked to refrain from drinking alcohol 24 h before each session, to avoid caffeine for 12 h before, not to do strenuous exercise 48 h before, to sleep at least 7 h the night before and to eat a regular meal 3 h before each session.

The specialized literature (Vaughan and Edwards, 2020; Hagyard et al., 2021) emphasizes the importance of considering practical sports experience and its relationship with IC. This relationship suggests that those with more sports experience may be better able to control their actions and maintain focus during sports activity. Therefore, to mitigate the variability associated with participants’ previous experiences, participants were organized into four groups based on their basketball practice in this study. Participants were differentiated according to their participation in federated competitions, their involvement in non-federated or recreational contexts, and the absence of basketball experience. The following groups were defined: High Practical Experience (HPE) for those with more than 10 years of experience in federated competitions; Medium Practical Experience (MPE) for those with 5 to 9 years of experience in federated competitions; Low Practical Experience (LPE) for individuals with practice in non-federated or recreational contexts, noting informal experience; and No Practical Experience (NPE) for those with no prior basketball experience, including both federated and non-federated contexts. Table 1 provides the profile and background information of the study participants.

**Table 1:** Profile and background information of the study participants.

Experience group	N Male/Female	Years of practical experience	Age (years)	Height (m)	Body mass (kg)	BMI (kg·m^−2^)
HPE	*N* = 10 7/3	11.20 ± 1.23	21.10 ± 3.99	1.77 ± 0.08	74.25 ± 7.81	23.68 ± 1.85
MPE	*N* = 10 7/3	7.00 ± 1.16	19.20 ± 1.69	1.80 ± 0.07	73.52 ± 9.08	22.58 ± 1.58
LPE	*N* = 14 11/3	2.29 ± 1.07	20.64 ± 2.74	1.77 ± 0.06	68.96 ± 9.86	21.95 ± 2.31
NPE	*N* = 10 6/4	–	20.40 ± 3.89	1.70 ± 0.08	66.15 ± 7.04	22.80 ± 1.42
General	*N* = 44 31/13	–	20.36 ± 3.13	1.76 ± 0.08	70.56 ± 8.96	22.68 ± 1.90

Data values are expressed as mean ± standard deviation. HPE, High Practical Experience; MPE, Medium Practical Experience; LPE, Low Practical Experience; NPE, No Practical Experience; N, Sample Size; M, Meters; Kg, Kilograms; BMI, Body mass index.

### 2.3 Design and procedure

#### 2.3.1 The rationale for the current design

The methodology used in the present study has been previously employed and documented (Gutiérrez-Capote et al., 2023). In that study, it was reported that players experienced an increase in perceived mental load and a decrease in motor performance under conditions involving practice variability and imposed restrictions. This previous study lays the methodological foundation for the current research, adopting a rigorous pre-experimental methodology to assess participants’ baseline cognitive characteristics and previous levels in basketball. This approach is aligned with recent contributions by Anzeneder et al. (2023) and Gutiérrez-Capote et al. (2024), who stress the importance of adapting the difficulty of sports interventions to the individual characteristics of each participant in order to avoid biases derived from the lack of consideration of their starting level. Thus, the challenges presented during the experimental phase must be adjusted to the participants’ previous experience. Building upon the foundation laid by the prior study, the experimental conditions were affirmed as suitable for addressing the objectives of this paper. The employed within-subject crossover design, integrating four factors, aimed to investigate the short-term impact of task restrictions during a basketball session on participants’ IC ability relative to their experience level. The following is a detailed summary of the methodology and its most relevant aspects.

#### 2.3.2 Study phases

##### 2.3.2.1 Informative and familiarization phase

This phase was carried out in a single session, lasting approximately one and a half hours, and its main objective was to provide the participants with a complete understanding of the study. At the beginning of this session, participants were given detailed information on the research objectives, the procedures to be followed, and the instruments to be used. Next, informed consent forms signed by the participants were handed out and obtained. Subsequently, critical data for the study were recorded, including information on the years of basketball experience of each player, which was essential for their grouping by level of experience. In addition, anthropometric data were collected, such as height and weight, measurements that would facilitate the subsequent calculation of body mass index (BMI; expressed in kg/m^2^). These measurements were taken with a measuring rod and a SECA 799 digital scale, with an accuracy of 0.1 kg (SECA, Germany).

##### 2.3.2.2 Pre-experimental phase

This phase consisted of two sessions conducted on different days, for which the participants were already grouped according to their levels of basketball experience. In one of the sessions, the IC capacity of the participants was assessed explicitly by applying the Flanker and Go-Nogo computer-based cognitive tests (detailed in Section 2.4.1, Cognitive test, of this manuscript). This session lasted approximately 30 min. In the other session, participants’ throwing ability, skill, and agility were assessed using basketball-specific tests, and their skill level was subsequently measured in a round-robin competition within each group. This session lasted approximately 1 h. A two-step cluster analysis was conducted to balance the level of competition between groups using the data collected from both sessions. This analysis allowed for pairing participants with similar abilities within each experience group, thus facilitating fair and equal competitions during the experimental sessions where participants would perform one-on-one (1 × 1) half-court basketball testing tasks. For more details on this phase, please refer to the work of Gutiérrez-Capote et al. (2023), specifically for the basketball tests used (*Physical Fitness and Sporting Ability* section, p. 8) and for the clustering analysis performed (*Clustering analysis section*, p. 5).

##### 2.3.2.3 Experimental phase

A conventional half-court 1 × 1 basketball task was used to perform the experimental sessions. In this, the attacking player started his attack phase one step behind the 6.75-meter line, directly in front of the basket, with his defender positioned in front of him. In the case of an offense-defense change of possession, the players would start from these same spaces. This task aims to maximize the number of baskets scored on offense and minimize those received on defense. Based on this task, two experimental sessions were carried out: one maintaining standard rules marked by the basketball regulations (Regular Practice, REG) and the other in which specific restrictions were applied to increase the difficulty (Restricted Practice, RES). In the RES modality, three types of restrictions were imposed to vary the usual game conditions: (A) Motor, restricting players to a maximum of three bounces to be able to move toward the basket in each offensive possession; (B) Temporal, restricting offensive possessions to 5 s to conclude their attack, in case of not finishing their attack action in that time they lost possession of the ball in favor of the defense; and (C) Spatial, restricting offensive displacements in a central area of the half court of 14 × 4.9 meters. The following measures were taken to ensure control of the experimental conditions: (1) Sessions were scheduled at the same time of day for each pair of participants, thus mitigating the effect of diurnal variations (Thun et al., 2015); (2) A required rest of at least 72 h between sessions was established for adequate recovery; (3) The same model of ball was used during the experimental sessions, with differentiated sizes for boys (size 7) and girls (size 6). In cases of mixed pairs, there was a researcher in charge of alternating the ball between attack phases to maintain equity; and (4) The intensity of the physical load was closely monitored and controlled by applying the training load methodology of Edwards (1993) (detailed in Section 2.4.2, Training load control, of this manuscript).

The experimental sessions began with the fitting of heart rate monitors for the participants. Next, the type of session they would conduct that day was disclosed, and any questions were clarified. Next, a researcher would lead a standardized 15-min warm-up (Scanlan et al., 2018). The sessions were structured into three practice blocks, each lasting 15 min. Each block included two 7-min 1 × 1 half-court basketball tasks, with a one-minute break between tasks. For the REG, the tasks remained unchanged throughout the three blocks.

In contrast, in RES, each block was dedicated exclusively to a specific restriction, varying the type of restriction applied in each block. The sequence of restrictions applied in each block in the RES was counterbalanced among all pairs. IC assessments were conducted using the computer-based Flanker and Go-Nogo cognitive tests at the end of each block, followed by a 3-min rest period between blocks. After the IC assessment of the third block, participants had a 15-min break before proceeding to another IC assessment. The sessions concluded with a 10-min cool-down and stretch. The duration of the experimental sessions was approximately 2 h.

### 2.4 Variables and instruments

#### 2.4.1 Cognitive test

Cognitive tests were conducted using the Psychological Experiment Building Language (PEBL, Version 2.1; Mueller and Piper, 2014). In the pre-experimental phase, participants were taken to a room designed to ensure a conducive environment, where computers were arranged so that participants could work comfortably and without distractions. Participants were instructed to sit comfortably, about 60 cm away from a 22″ computer screen with a black background, running the Windows operating system. These computers were equipped with a mouse, placed next to the side of the participant’s dominant hand. In the case of the experimental sessions, additional measures were implemented to avoid mutual peer influence.

Consequently, the computers were set up in isolated spaces within the hall, and two researchers closely supervised each participant, ensuring the test ran smoothly. In both phases, the order of the computer tests was counterbalanced between pairs and all study participants. The tests used were:

Go-Nogo task: The task assesses response inhibition (Bezdjian et al., 2009). Participants were presented with a screen divided into four quadrants to provide a rapid and accurate motor response through a right-click when presented with a target letter. Subsequently, a single letter (“P” or “R”) was displayed in one of the quadrants for a duration of 500 ms, with a 1,500 ms interval between stimuli. The experiment consisted of two distinct phases. In the initial phase, participants were instructed to respond when the letter “P” was displayed and to withhold their response when “R” appeared. In the subsequent phase, the instructions were reversed, requiring participants to respond to the letter “R” and withhold their response when “P” was presented. Each phase comprised 10 practice trials and 50 experimental trials, with a distribution of 40 trials involving target letters (e.g., P-Go) and 10 trials involving non-target letters (e.g., R-Nogo). A target-to-non-target ratio of 80:20 was maintained. The behavioral performance of the task was analyzed by: (1) the number of correct responses to the target letter (Go) (hits); (2) omission errors, which are when responding to the letter Go is missed; (3) commission errors, which refer to responding incorrectly to the letter Nogo; and (4) correct refusals to the letter Nogo. In addition, reaction time (RT), RT variability in responses to the letter Go, and the cost associated with switching between the two parts of the test were also assessed and calculated for each participant. The task duration was approximately 6 min.

Flanker task: The task assesses interference control (Eriksen and Schultz, 1979; Stins et al., 2007). In this task, participants were required to respond to the direction of a white arrow positioned in the center of the computer screen. To execute this task, participants had to press the left shift button with their left index finger when the arrow pointed to the left (“<”) and the right shift button with their right index finger when the arrow pointed to the right (“>”). The task involved four distinct flanking conditions: (1) Congruent: In this condition, all arrows pointed in the same direction (“< < <”or “> > > ”); (2) Incongruent: This condition involved arrows pointing in different directions (“< < > < <” or “> > < > >”); (3) Neutral: Under this condition the central arrow is displayed alone (“<” or “>”); and (4) Dash: In this case, the central arrow lacks any distracting stimulus (“-- < −-” or “-- > −-”). Each testing session consisted of a block of 90 trials, including an initial set of 10 practice trials. These were followed by 20 congruent trials, 20 incongruent trials, 20 neutral trials, and 20 dash trials, presented randomly within each block. Each experimental trial commenced with a 500 ms presentation of a white fixation cross in the computer screen’s background. Subsequently, a stimulus was displayed for 800 ms, with a 1,000 ms interval between stimuli. The primary variables analyzed included accuracy and average RT for each trial type. The duration of the task was approximately 6 min.

#### 2.4.2 Training load control

It was sought to ensure that participants performed the 1 × 1 tasks within a high-intensity range commensurate with the demands of real basketball play. To this end, we relied on the results presented by Stojanović et al. (2018). In that systematic review, it was reported that maximum heart rate (HRmax) values in men and women during active participation in games fluctuated, spanning a range between 81.8 and 94.6%. By these findings, it was established that participants should perform in a range of 80–90% of their HRmax. Furthermore, this working range was considered appropriate to address the study’s objectives, as cognitive performance may be affected by physical and emotional fatigue associated with high-intensity exercise (Barnes and Van Dyne, 2009). To achieve this objective, we employed Edwards’ training load (Edwards, 1993) method, widely recognized in the basketball context, which allows us to evaluate both the internal training load (Manzi et al., 2010; Conte et al., 2016; Sansone et al., 2019; Camacho et al., 2021) and correlate it with the external one (Scanlan et al., 2014). This approach classifies exercise intensity into five heart rate (HR) zones about the percentage of HRmax (50–60% HRmax = 1, 60–70% HRmax = 2, 70–80% HRmax = 3, 80–90% HRmax = 4, 90–100% HRmax = 5). We calculated the percentage of each participant’s HRmax using the formula 220 minus age (Fox and Naughton, 1972). Subsequently, we evaluated the training load for each participant, which allowed us, during the experimental sessions, to monitor in real-time the participants’ HR using a Polar Pulsar RS800CX sensor (Polar Electro Oy, Kempele, Finland) and ensure that they were working within the optimal zone.

### 2.5 Statistical analysis

The data summaries, which include the mean and standard deviation, were computed for the entire sample set. Subsequently, linear mixed model analysis (LMMs) was employed, which is more appropriate in studies with repeated measures design because it considers specific patterns at the individual level, including them as random factors when correlations between the conditions of an experiment are likely to exist (Meteyard and Davies, 2020). This analysis was performed to investigate: (1) verify that the training load objective was met in both experimental conditions; (2) the impact of each experimental condition on cognitive performance (IC); (3) disparities between experimental conditions in IC; and (4) in cases where significant differences were observed, we examined whether they were mediated by basketball experience by incorporating experience groups into the session model (i.e., the model that encompassed the distinctions between the two sessions). We then evaluated the contribution of experience to model fit by comparing these models against the session model. This hierarchical analysis allowed us to determine if the increase in the proportion of explained variability attributed to the independent variable of interest (either experience or cognition) relative to a model without that independent variable justified the added complexity of the model, indicating the presence of a moderating effect.

LMMs are an extension of linear models that incorporate random effects into the linear predictor term within the regression framework. They enable the modeling of dependence structures among dependent variables, particularly in longitudinal or repeated-measures data. Model selection was based on the Akaike information criterion (AIC) and the χ^2^ test to determine whether the new models provided a better fit than the session model, implying an interaction between the included variables. Furthermore, we assessed effect sizes using *R*^2^ by Cohen’s guidelines (Cohen, 1988): Weak (0.02), moderate (0.13), substantial (0.26). The model with the lower AIC was considered a better fit, signifying the existence of an interaction between the variables. The construction of each model can be observed in the first paragraph of each results section. Statistical significance was set at *p* < 0.05.

LMMs analyses used the ‘lmer’ function from the ‘nlme’ package in R (Pinheiro et al., 2012). Prior to analysis, all quantitative predictors were standardized and centered at zero. Effect sizes were calculated using the Nakagawa–Schielzeth approach (Nakagawa and Schielzeth, 2013).

## 3 Results

### 3.1 Descriptive statistics and training load check

A descriptive study of the performance variables of IC was conducted, revealing in Table 2 the means and standard deviations for each variable. As for the training load, as expected, no significant differences were observed between the blocks because it was ensured that all participants scored a 4 on Edward’s scale.

**Table 2:** Descriptives of inhibitory control performance between sessions.

	Flanker incongruent RT (ms)	Flanker congruent RT (ms)	Flanker incongruent ACC (%)	Flanker congruent ACC (%)	Go-Nogo RT (ms.)	Go-Nogo total ACC (%)	Nogo trials ACC (%) ACC (%)
Baseline	455(±42.1)	417(±45.7)	0.93(±0.05)	0.98(±0.03)	458(±55.1)	0.92(±0.03)	0.70(±0.12)
*Regular practice*							
Block 1	415(±38.2)**	385(±36.2)**	0.90(±0.09)*	0.97(±0.06)	396(±33.9)**	0.91(±0.04)	0.67(±0.15)*
Block 2	411(±44.0)**	377(±38.8)**	0.88(±0.10)**	0.96(±0.05)	389(±39.1)**	0.90(±0.04)	0.66(±0.16)**
Block 3	404(±42.4)**	367(±35.1)**	0.89(±0.11)**	0.97(±0.04)**	385(±29.8)**	0.91(±0.04)	0.67(±0.15)**
Recovery	415(±48.8)	379(±36.4)	0.90(±0.09)**	0.96(±0.04)	388(±32.2)**	0.90(±0.04)	0.66(±0.14)*
*Restricted practice*							
Block 1	470(±41.3)**	426(±38.9)**	0.85(±0.12)*	0.96(±0.04)	472(±47.3)**	0.90(±0.04)	0.59(±0.19)*
Block 2	466(±42.7)**	426(±61.3)**	0.79(±0.15)**	0.97(±0.05)	474(±46.1)**	0.90(±0.04)	0.55(±0.21)**
Block 3	465(±48.6)**	431(±68.0)**	0.76(±0.15)**	0.93(±0.06)**	477(±53.1)**	0.90(±0.04)	0.55(±0.19)**
Recovery	416(±49.3)	379(±33.8)	0.83(±0.13)**	0.96(±0.04)	434(±53.5)**	0.90(±0.04)	0.58(±0.18)*

Means and standard deviations are presented. RT, response time; ACC, accuracy; ms, milliseconds; significant differences between experimental conditions were marked with: *p < 0.05; **p < 0.001.

### 3.2 LMMs–experimental condition effect on inhibitory control

The primary outcome measure was IC, assessed through Flanker and Go-Nogo tasks at baseline, across three blocks, and during the recovery phase. Each participant was treated as a random factor in each experimental condition (e.g., 1|participant). To ascertain whether there were changes in IC during the session, we compared baseline to the first block, analyzed differences among blocks, and evaluated differences between the last block and the recovery phase.

During the REG session, a significant reduction in RT was observed in the Flanker task for both Congruent and Incongruent trials between baseline and block 1 (Incongruent: *p* < 0.001; Congruent: *p* = 0.025). No significant differences in RT were found between subsequent blocks or the recovery phase. Additionally, no significant differences in accuracy were observed at any phase. Similar results were obtained in the Go-Nogo task.

**Figure 1:**
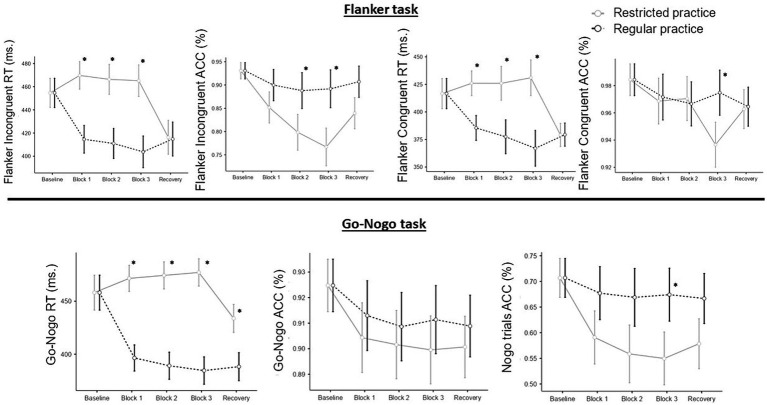
Effect of both experimental conditions in Flanker and Go-Nogo tasks. RT, response time; ACC, Accuracy. Significant differences between type of practice in each block are marked with “*”.

In the RES session, no significant results were observed in RT. However, a significant decrease in accuracy was noted between baseline and block 1 in both the Flanker and Go-Nogo tasks (*p* = 0.002). These results are graphically illustrated in Figure 1.

### 3.3 LMMs – differences among sessions

Each participant’s IC performance was the principal outcome variable of interest. Each participant was treated as a random factor, and the models involved the inclusion or exclusion of the session variable as a fixed factor. The following section presents the results, which are also visually depicted in Figure 1.

In both Congruent and Incongruent trials during the Flanker task, participants exhibited a significant deceleration in RT across all blocks within the RES session, compared to the REG session. With respect to accuracy, a notable decrease was observed in Incongruent trials during the first two blocks of the RES session. Block 3 also revealed a reduction in accuracy for Congruent trials. The sole discrepancy noted during the recovery phase was the lowered accuracy in Incongruent trials within the RES session.

In the Go-Nogo task, RT consistently demonstrated lower values in RES than those in REG across all blocks, including the recovery phase. As for accuracy, participants in the RES session committed more omission errors (pressing in Nogo trials), and this pattern was observed across all blocks, including the recovery phase.

Comprehensive details of the model results are presented in Table 3 and visually in Figure 1.

**Table 3:** Linear mixed models results checking differences between sessions.

Task	Block	Measure	Condition	Model	AIC	*p*-value	*R* ^2^
*Flanker*	1	RT	Incongruent	Without session	254.72	–	–
				Including session	219.45	<0.001	0.33
			Congruent	Including session	233.69	<0.001	0.23
	2		Incongruent	Including session	226.02	<0.001	0.29
			Congruent	Including session	238.59	<0.001	0.18
	3		Incongruent	Including session	222.01	<0.001	0.31
			Congruent	Including session	229.79	<0.001	0.26
	1	Accuracy	Incongruent	Without session	250.94	–	–
				Including session	246.99	0.014	0.04
	2		Incongruent	Without session	254.68	–	–
				Including session	245.30	<0.001	0.11
	3		Incongruent	Without session	253.77	–	–
				Including session	232.04	<0.001	0.18
			Congruent	Without session	253.53	–	–
				Including session	242.28	<0.001	0.11
	Recovery		Incongruent	Without session	254.72	–	–
				Including session	248.52	0.004	0.08
*Go-Nogo*	1	RT	–	Without session	254.72	–	–
			–	Including session	202.49	<0.001	0.33
	2		–	Including session	195.06	<0.001	0.34
	3		–	Including session	188.09	<0.001	0.37
	Recovery		–	Including session	235.14	<0.001	0.12
	1	Accuracy	–	Without session	247.72	–	–
			–	Including session	240.19	0.002	0.33
	2		–	Without session	254.71	–	–
			–	Including session	248.83	0.005	0.07
	3		–	Without session	252.57	–	–
			–	Including session	238.79	<0.001	0.39
	Recovery		–	Without session	253.51	–	–
			–	Including session	247.45	0.004	0.17

RT, Response time; AIC, Akaike information criterion.

### 3.4 LMMs – checking the moderation by experience

New models were constructed to assess whether participants’ experience levels influenced the disparities across sessions, incorporating the four experience groups and their respective IC performance. No statistically significant differences were detected at baseline. In the Incongruent trials of the Flanker task, it was observed that greater experience led to faster performance in block 1 (*p* = 0.056) and enhanced accuracy across all blocks, particularly in block 3 and the recovery block.

**Figure 2:**
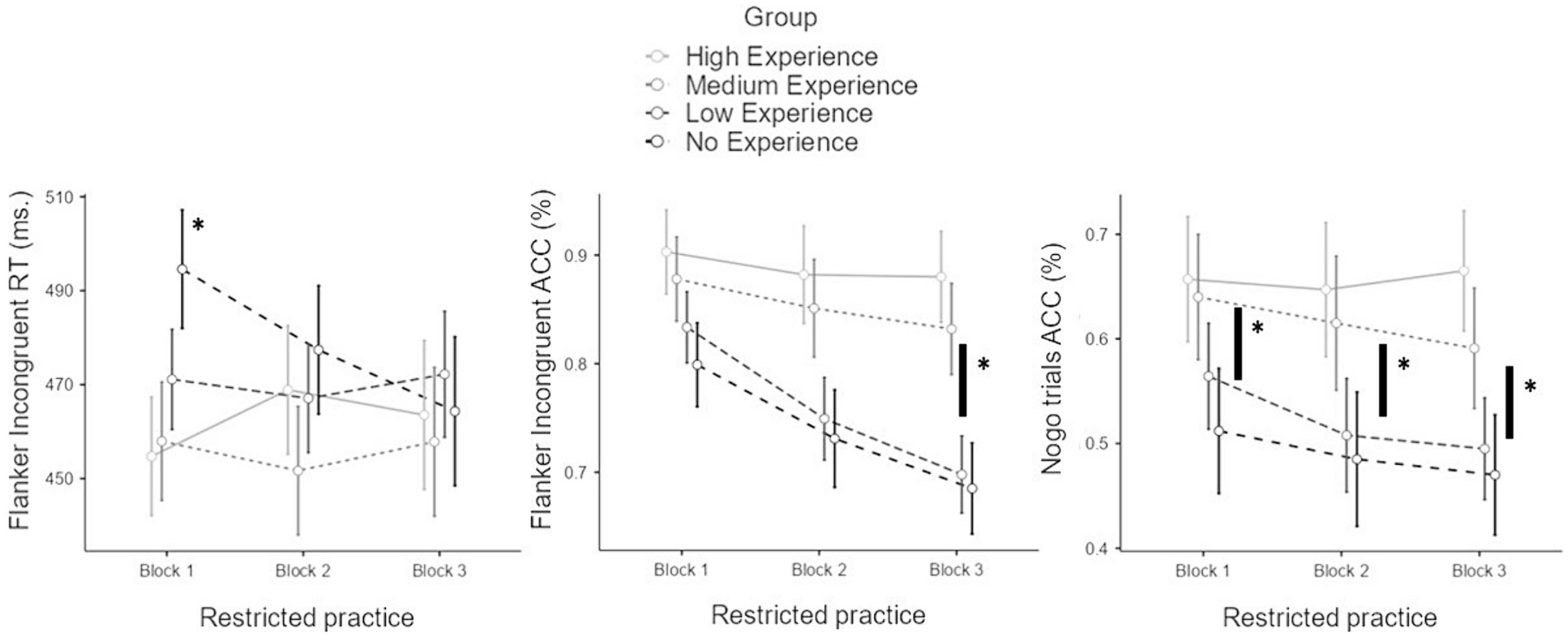
Moderation by experience in the response time and accuracy of the incongruent trials of the Flanker task and accuracy in the Nogo trials of the practice with restrictions session. RT, response time; ACC, Accuracy. Significant differences between groups of experience in each block are marked with “*”.

In the Go-Nogo task, more experience was associated with fewer omission errors during blocks 1, 3, and the recovery phase. Detailed model results are presented in Table 4 and visually depicted in Figure 2.

**Table 4:** Linear mixed models results checking moderation by experience.

Task	Block	Model	AIC	*p*-value	*R* ^2^
*Flanker (Incongruent Trials)*	Block 1	Model without experience	129.85	–	–
		Model including experience	127.50	0.034	0.21
	Block 2	Model including experience	128.65	0.056	0.19
	Block 3	Model including experience	113.25	<0.001	0.43
	Recovery Block	Model without experience	−49.41	–	–
		Model including experience	−56.19	0.005	0.49
*Go-Nogo*	Block 1	Model without experience	129.85	–	–
	Block 1	Model including experience	122.19	0.003	0.30
	Block 3	Model including experience	121.66	0.002	0.31
	Recovery Block	Model including experience	119.83	0.001	0.34

RT, Response time; AIC, Akaike information criterion.

## 4 Discussion

This study aimed to examine the acute effects of sports practice on the inhibitory control of healthy young individuals. The findings revealed that altering task complexity by imposing restrictions (motor, temporal, and spatial restrictions) had an acute impact on participants’ inhibitory capability. Engaging in specific and routine basketball training exercises immediately enhanced the capacity for inhibition. Conversely, engaging in more challenging tasks led to a decline in this capacity. Our study revealed that players’ inhibitory control was influenced by the difficulty level of the training tasks, resulting in contrasting effects depending on the dose. These outcomes support our first hypothesis, suggesting that the level of complexity in the tasks undertaken by players determines whether there will be a positive or negative impact on their response inhibition and interference control.

When participants performed Regular Practice (REG), the result replicates the beneficial effect observed in ecological contexts in inhibitory control following a single sports bout (complex skills) compared to simple exercise without cognitive requirements (Takahashi and Grove, 2019, 2020).

According to our results, the complexity of a task influences cognitive load. Specifically, cognitively challenging exercise requiring bodily coordination demands additional cognitive effort. The more complex the task, the more significant cognitive effort is required from the individual (Paas et al., 2003). Our findings of reduced reaction time in both cognitive tasks after REG compared to the baseline state suggest that executive control was engaged during REG and that the ability to activate this control extended into the post-game period. However, the effect only persisted during the initial block. Beyond this period, reaction time levels returned to their baseline state. The acute effects of sports, involving complex motor tasks and high cognitive demands, may benefit from stimuli that pre-activate brain regions that control higher-order cognitive processes, potentially enhancing executive function (Tomporowski et al., 2015). Consequently, as evidenced by inhibitory control results, participants could process contextual information more efficiently and rapidly (Paschen et al., 2019).

On the other hand, adverse effects were also observed in the experimental condition of Restricted Practice (RES). Engagement in more challenging tasks with higher cognitive load, achieved through the imposition of restrictions (i.e., motor, temporal, and spatial), led to a decrease in inhibitory control. Specifically, unlike the REG condition, our findings regarding response accuracy in the Go-Nogo and Flanker tasks after practice, showed a significant player’s accuracy reduction compared with baseline performance. This deterioration is frequently observed in studies that have tailored specific tasks to accommodate the demands of various sports, thereby investigating the repercussions of mental fatigue on cognitive abilities (Filipas et al., 2021; Fortes et al., 2023). For instance, Slimani et al. (2018) demonstrated that mental fatigue had a detrimental effect on selective attention in concentration tasks. Mental fatigue reduces the ability to suppress irrelevant information, leading participants to increasingly base their decision-making on irrelevant information, resulting in lower response accuracy (Faber et al., 2012).

As a task becomes more complex, it may demand greater attentional resources from the individual, leading to increased mental effort (Dudley et al., 2021). However, there is a limit beyond which the increase in cognitive load can overwhelm the information processing system’s capacity, negatively affecting motor performance and the ability to handle additional tasks or respond to unforeseen situations (Guadagnoli and Lee, 2004). Therefore, attempting to execute the task effectively results in an immediate decrease in cognitive performance (Salihu et al., 2022).

These effects can be elucidated by the relationship between the practice of physical-cognitive exercise and enhanced top-down processing in the prefrontal cortex (Lucia et al., 2023). Physical-cognitive tasks drive inhibition by bolstering the modulation of neural processes involved in conflict monitoring (Patelaki et al., 2023). This processing is associated with a proactive type of cognitive control, whereas bottom-up processing is linked to a reactive type of control (Braver, 2012). Attention control studies suggest that difficulties in proactive control may exist in different environments and populations (Li et al., 2017). In conditions of high cognitive load, deactivation has been observed in cortical areas associated with the cognitive control network that supports top-down behavior control (Mansouri et al., 2009). On the contrary, when examining acute effects during physical tasks with cognitive demands, participants who achieve benefits in their inhibitory capacity demonstrate a more proactive cognitive strategy (Patelaki et al., 2023).

The overall task context, including its demands and characteristics, is crucial in modulating proactive or reactive control strategies (Skau et al., 2021). For instance, the readiness potential (also called the Bereitschaftspotential or the premotor potential), a component of event-related potentials (ERP) extensively investigated in proactive control, is influenced by the complexity of the motor task (Colebatch, 2007). Increasing task difficulty due to increased temporal or spatial constraints may hinder proactive motor control by diminishing the player’s capacity for interference anticipation (Braver et al., 2007). The Flanker and Go-Nogo tasks necessitate proactive control that could assist participants in foreseeing conflict following Incongruent or Nogo trials and preparing to halt motor responses (Liebrand et al., 2018). The extra mental effort exerted during RES could imply a shift in the type of cognitive control. In this context, it is crucial to recognize that fatigued individuals tend to employ reactive cognitive control (Brick et al., 2016; Skau et al., 2021). Therefore, our speculation is substantiated by this observation, emphasizing that subjects, when fatigued, tend to opt for reactive control over proactive control.

To conclude, the player’s response would be influenced by two specific thresholds: one promoting proactive control and another impeding it. Surpassing the first threshold would enhance cognitive performance, as benefits would be activated by stimulating physical and cognitive activity with a moderate or vigorous load. However, sustaining this load over time could lead to volume-induced fatigue or increased difficulty, representing the second threshold. Surpassing this second threshold would induce an acute deterioration in cognitive performance. Thus, it is crucial to distinguish between the threshold that fosters benefits and the threshold that leads to deterioration, providing a clearer understanding of the relationship between the two. As outlined, these thresholds could be susceptible to environmental changes, such as tasks involving both physical and cognitive demands. Initially, both physical and cognitive load during RES may facilitate a shift from a more reactive to a more proactive control (Kamijo and Masaki, 2016), reverting to a reactive state again due to increased environmental demands surpassing proactive control’s resources, as it is more costly and much more challenging to implement. This phenomenon has been observed in populations with fewer resources, such as older adults or children (Chevalier et al., 2012).

Finally, this study analyzed the possible influence of the players’ sports experience on the relationship between the task’s difficulty and their subsequent cognitive performance. The results showed the moderating effect of playing experience in attenuating the effect of increasing task difficulty on inhibitory control. Those players with more experience perceived fewer mental demands (Gutiérrez-Capote et al., 2023), translating into better results in their cognitive performance in the RES. Within the context of the optimal challenge framework, it is established that the functional difficulty of a task, in addition to being affected by experience and practice conditions, also depends on individual processing characteristics (Guadagnoli and Lee, 2004). This idea finds support in various research on elite athletes, which indicates that highly skilled athletes exhibit more efficient inhibitory control than semi-professional and amateur-level athletes (Hagyard et al., 2021). Consequently, a close relationship between the players’ cognitive and athletic performance must be considered. However, future interventional research should check the direction of such a relationship. Although our results claim to be cautious, it could be that for the most experienced players the perception of the mental load required is lower when performing tasks, and, therefore, they could need fewer cognitive resources to perform them (Yu et al., 2019). Understanding this directional relationship could help design strategies to improve athletes’ cognitive performance.

The mixed results on inhibitory control offer a novel perspective to interpret the dose–response effects on cognitive performance produced by increasing nominal task difficulty. However, these results should be treated with caution. Studies showing positive effects of physical exercise (PE) on inhibitory control usually compare entirely different activities (Benzing et al., 2016) and suffer from controlling for the degree of physical and mental task load through some external measure. On the other hand, studies showing the adverse effects of PE practice are limited, and it is not easy to establish a causal relationship between the results (see Egger et al. (2018)). Moreover, in their interventions, the definition of the quantitative aspects of the tasks is very disparate, and individualized adaptation was not performed.

However, the value of the results reveals the need for such an adjustment. While these environments may initially result in less adaptive behavior with more errors in the short term, challenging and practice-friendly training environments have been found to facilitate learning in the long term (Frömer, 2016). These early-stage errors could provide an essential means of stimulating an individual’s adaptive capacity (Turakhia et al., 2021). Unfortunately, the design of our study does not allow us to contrast whether the acute adverse effect of cognitively more demanding practice (RES) on cognitive performance could revert to long-term benefits if these stimuli were repeated over time. Future studies should investigate the relationship between the acute and chronic effects to draw reliable conclusions.

## 5 Limitations

Despite finding evidence on the positive and negative effects of complexity in a task-restricted session on inhibitory control, the present study has some limitations. First, the sample size in each experience group is small and, in addition, we decided to present the results of the male and female participants together. This limitation highlights the need for a larger number of participants and the need for additional studies that can replicate these findings. However, it is essential to note that linear mixed model analysis (LMMs) was chosen for two main reasons to control for this limitation. First, it is the most appropriate approach for studies with multiple repeated measures (Meteyard and Davies, 2020). Second, this type of analysis can mitigate the limitations of having a small sample size. Since mixed effects analysis involves comparing models based on their AIC, this bears some similarity to Bayesian principles (Bozdogan, 1987). Moreover, Bayesian methods can provide meaningful results even with small samples by integrating prior information and updating beliefs with the arrival of new data. This feature is precious in contexts where data is scarce or costly (Schmid and Stanton, 2019).

Second, we tried to homogenize the level of difficulty that players would experience by facing, within each group, participants with similar skill levels in the 1 × 1 tasks. However, in the groups of less experienced players, a more in-depth evaluation would have been necessary due to the presence of participants coming from other sports disciplines, who could have had some advantage due to the transfer of learning. Finally, the availability of studies that address PE or sports interventions and report adverse effects is minimal. This paucity of research that comprehensively details and analyzes the potential adverse effects of physical activity poses a challenge to obtaining enlightening conclusions.

## 6 Future lines research

Future research should study the effect of learning in this type of practice. In addition, it would be interesting to study whether the short-term benefits or deterioration of cognition due to sports practice (acute effect) are reflected in the long term (chronic effect). For this purpose, it would be advisable to develop longitudinal studies in which the training loads of each session would be monitored (Perrey, 2022). In this way, evaluating the true long-term impact of stimuli that generate a post-session cognitive benefit would be possible concerning those that momentarily deteriorate cognitive performance. This would open the possibility of analyzing the cognitive development and skills acquired through sports practice, manifesting in allocating resources to improve the athlete’s adaptive capacity. Exploring how practice conditions that generate greater cognitive and emotional involvement influence their subsequent cognitive performance is also interesting.

## 7 Conclusion and practical applications

Our findings provide a different perspective on the interaction between the complexity of an acute exercise task and subsequent cognitive performance outcomes. By introducing restrictions that modify the complexity, participants faced additional challenges, resulting in a temporary deterioration in the player’s inhibitory capacity, persisting even after a 15-min recovery period. This study highlights the importance of initial levels of sports experience as significant moderators of the observed effects, thus providing a more accurate understanding of the complex interplay between exercise-induced cognitive load and its impact on cognitive performance. These aspects are essential to enhance the development of inhibitory control and test the tasks’ functionality. In this sense, these approaches will help to adapt the tasks’ difficulty according to each athlete’s sports experience, providing an optimal challenge that favors the cognitive and adaptive growth of the players (Guadagnoli and Lee, 2004).

In terms of practical applications, these perspectives can benefit professionals in various fields, such as sports, education, recreation, or wellness, since the practitioner’s observation of performance dynamics on a training task can help identify the difficulty level to stimulate the athletes’ performance conservation (practice to maintain) or the performance improvement (practice to learn).

## Data availability statement

The data that supports the findings of this study and the R code used are available in Figshare at https://doi.org/10.6084/m9.figshare.25772268.v1. The data is openly accessible and can be viewed online for free.

## Ethics statement

The studies involving humans were approved by Ethical Committee on Human Research of the University of Granada (3616/CEIH/2023). The studies were conducted in accordance with the local legislation and institutional requirements. The participants provided their written informed consent to participate in this study.

## Author contributions

AG-C: Conceptualization, Data curation, Investigation, Methodology, Writing – original draft, Writing – review & editing. IM: Conceptualization, Data curation, Formal analysis, Investigation, Methodology, Writing – review & editing. FA: Conceptualization, Data curation, Funding acquisition, Investigation, Methodology, Supervision, Writing – original draft, Writing – review & editing. ET: Data curation, Investigation, Writing – review & editing. JJ-M: Data curation, Investigation, Writing – review & editing. DC: Conceptualization, Funding acquisition, Investigation, Methodology, Project administration, Resources, Supervision, Writing – original draft, Writing – review & editing.
